# Multimodality Imaging of a Silent Killer

**DOI:** 10.14797/mdcvj.1073

**Published:** 2022-03-04

**Authors:** Ola Abdelkarim, Yehia Saleh, Summit Pandat, Mouaz Al-mallah

**Affiliations:** 1 Houston Methodist DeBakey Heart & Vascular Center, Houston Methodist Hospital, Houston, Texas, US

**Keywords:** cardiomyopathy, myocardial infarction, cardiac magnetic resonance imaging, cardiac computed tomography, cardiac fluorodeoxyglucose-positron emission tomography

## Abstract

**CME CREDIT**

Earn free AMA PRA Category 1 Credit^TM^ by reading this case and reviewing the video quizzes embedded. Then follow the link to obtain CME credit.

## Clinical presentation

**Figure 1:**
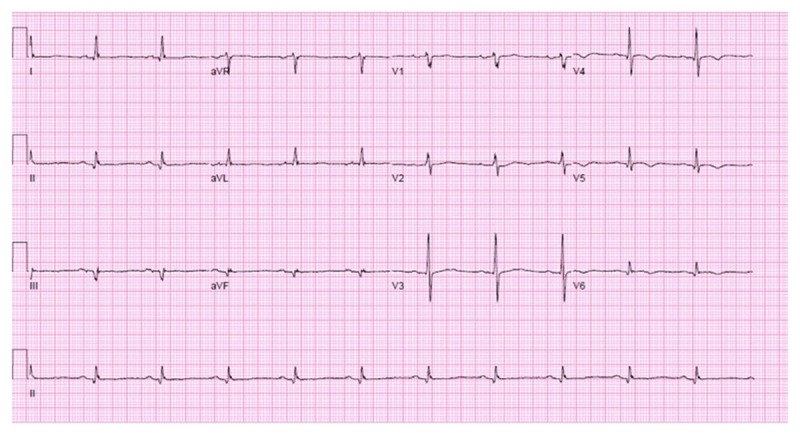
Electrocardiogram of patient presenting with exertional dyspnea and fatigue.

A 60-year-old man had undergone mechanical mitral valve replacement in 2012 due to mitral valve prolapse complicated by infective endocarditis. He has a history of atrial fibrillation, for which he underwent pulmonary vein isolation with mitral isthmus ablation in March 2020. He presented 1 year later with a 4-week history of exertional dyspnea and fatigue. Vital signs were within normal limits. Physical exam was remarkable for a mechanical S1 click on cardiac auscultation. His medication regimen included coumadin, aspirin 81 mg daily, amiodarone 200 mg daily, and rosuvastatin 10 mg daily. Laboratory workup was unremarkable except for a slightly subtherapeutic international normalized ratio (INR) of 2.3 and a mildly elevated brain natriuretic peptide (BNP) of 150 pg/mL. An electrocardiogram (ECG) is shown in ***Figure 1***, and chest x-ray (CXR) showed a slightly increased cardiothoracic ratio with clear lung fields.

## QUESTION 1

**What is the most likely cause of dyspnea in this patient?**

- Myocardial infarction
- Prosthetic valve dysfunction
- Congestive heart failure
- Pulmonary embolism
- Requires more investigation

**Question 1:** Consider the options and find the answer in this video quiz, also at *https://youtu.be/5BQTEHbMlew*.

12-lead ECG (***Figure 1***) showed a normal sinus rhythm with Q waves in leads II and III, and aVF and inverted T wave in leads V4, V5, and V6. Theoretically, all of the above are possible causes of dyspnea in this patient; however, none of the aforementioned answers will fit perfectly with the clinical presentation. Although the patient presents with subtherapeutic INR and mildly elevated BNP, the patient is euvolemic on exam, CXR does not show signs of congestion, and he had crisp mechanical heart sounds that make mitral valve prosthesis dysfunction and congestive heart failure unlikely. ECG showed Q waves in the inferior territory suggesting an old myocardial infarction, but the patient denies any history of known myocardial infarction or chest pain. Additionally, the troponin was negative. Pulmonary embolism would be highly unlikely in an anticoagulated patient. To further evaluate his symptoms, transthoracic echocardiography (TTE) was done (***Videos 1^2^,3***).

**Video 1:** Play the video to see what the transthoracic echocardiogram revealed, also at *https://youtu.be/WHHURP04pNM*.

**Video 2:** Play the video to see what the transthoracic echocardiogram revealed, also at *https://youtu.be/n4PSr3e-18Q*.

**Video 3:** Play the video to see what the transthoracic echocardiogram revealed, also at *https://youtu.be/BHd8hmcOibY*.

## Question 2

**What is the diagnosis?**

- Pericardial effusion
- True ventricular aneurysm
- Ventricular pseudoaneurysm
- Loculated pleural effusion

**Question 2:** Consider the options and find the answer in this video quiz, also at *https://youtu.be/HYEAt-GqSPU*.

TTE showed a mildly impaired left ventricular (LV) systolic function (***Video 1***) with an ejection fraction (EF) in the 40% to 45% range and a lateral wall pseudoaneurysm (***Videos 2^3^***). ***Video 2*** shows a narrow neck opening from the posterolateral wall to the aneurysm, while ***Video 3*** demostrates free passage of the ultrasound contrast from the LV to the pseudoaneurysm. Doppler recordings indicated normal function of the mechanical mitral valve (not shown).

**Figure 2:**
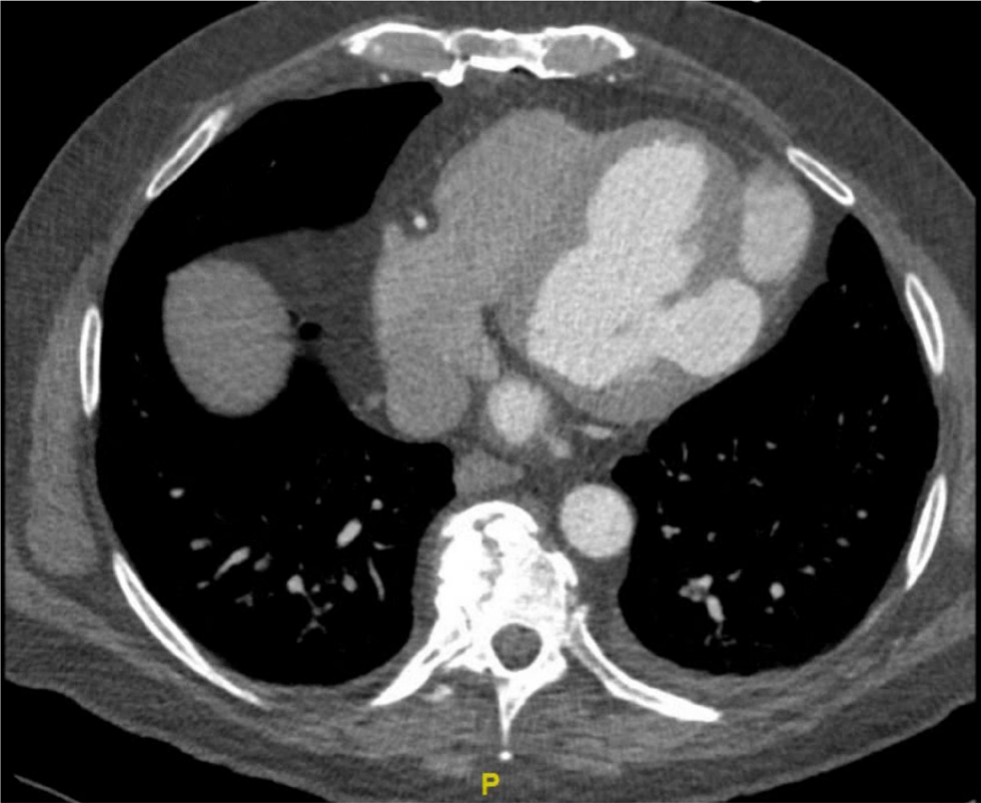
Computed tomography showing a large bilobed posterolateral left ventricular pseudoaneurysm.

A cardiac computed tomography (CT) scan (***Figures 2^3^; Video 4***) confirmed the TTE findings and demonstrated a large bilobed posterolateral LV pseudoaneurysm measuring up to 7.8 cm in length. Coronary CT angiography showed mild coronary atherosclerosis but no significant stenosis.

**Figure 3:**
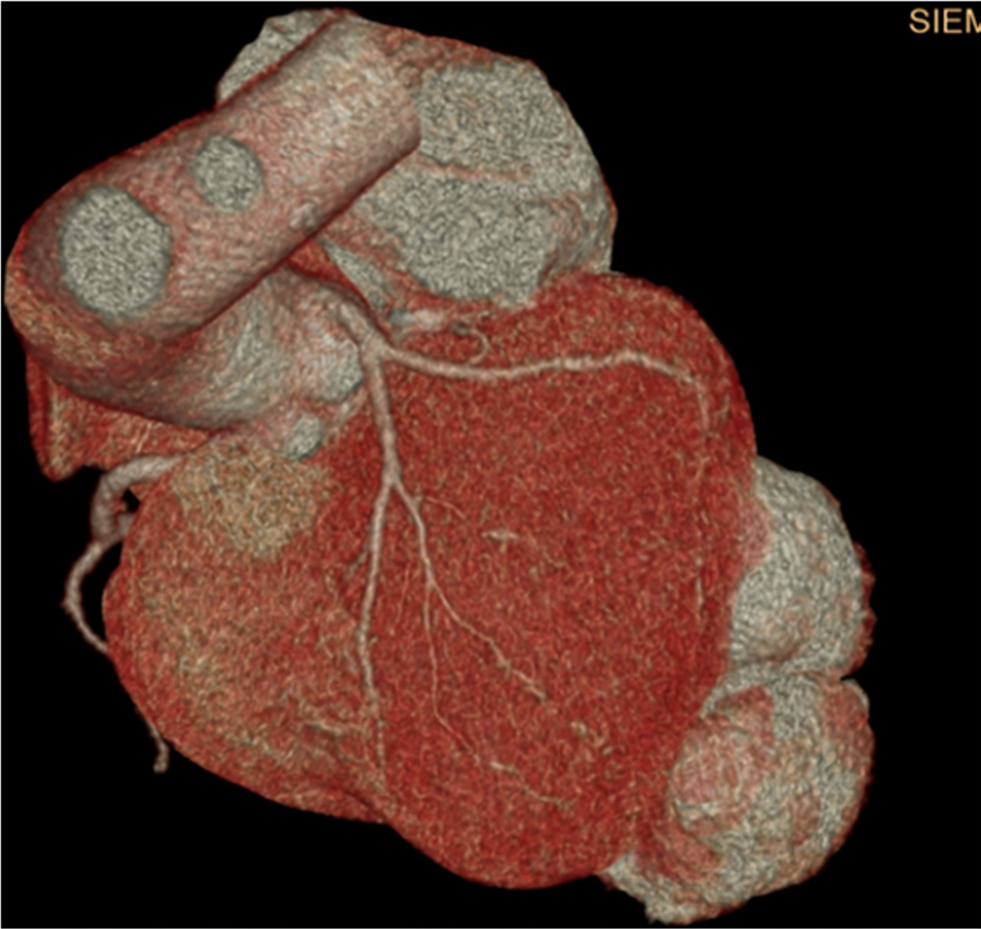
3-dimensional reconstructed computed tomography showing a large bilobed posterolateral left ventricular pseudoaneurysm.

**Video 4:** Computed tomography showing a large bilobed posterolateral left ventricular pseudoaneurysm, also at *https://youtu.be/ks6FDJgg41g*.

## QUESTION 3

**What would help in revealing the etiology of the ventricular pseudoaneurysm?**

- Cardiac magnetic resonance imaging (CMR)
- Cardiac fluorodeoxyglucose positron emission tomography (FDG-PET)
- Coronary angiography
- All of the above

**Question 3:** Consider the options and find the answer in this video quiz, also at *https://youtu.be/X4VrGOVJNbk*.

Cardiac fluorodeoxyglucose-positron emission tomography (FDG-PET) is a noninvasive imaging modality that differentiates normal myocardium from scarred myocardium by lack of FDG uptake. The patient underwent FDG-PET and it did not show FDG uptake in the pseudoaneurysm (***Video 5***). There was no extra cardiac FDG uptake to suggest inflammation. Cardiac magnetic resonance (CMR) can define anatomy, measure LV volume, and assess myocardial viability. CMR showed a basal–mid-lateral wall myocardial infarction with contained rupture resulting in a large (8.1 × 3.9 cm) LV pseudoaneurysm (***Video 6***). Interestingly, coronary angiography was normal.

**Video 5:** Cardiac fluorodeoxyglucose-positron emission tomography did not show uptake of fluorine-18-deoxyglucose in the pseudoaneurysm, also at *https://youtu.be/_Yfb9SBNDYI*.

**Video 6:** Cardiac magnetic resonance shows a basal–mid-lateral wall myocardial infarction with contained rupture resulting in a large (8.1 × 3.9 cm) left ventricular pseudoaneurysm, also at *https://youtu.be/zcRz8J8gqxs*.

**Figure 4:**
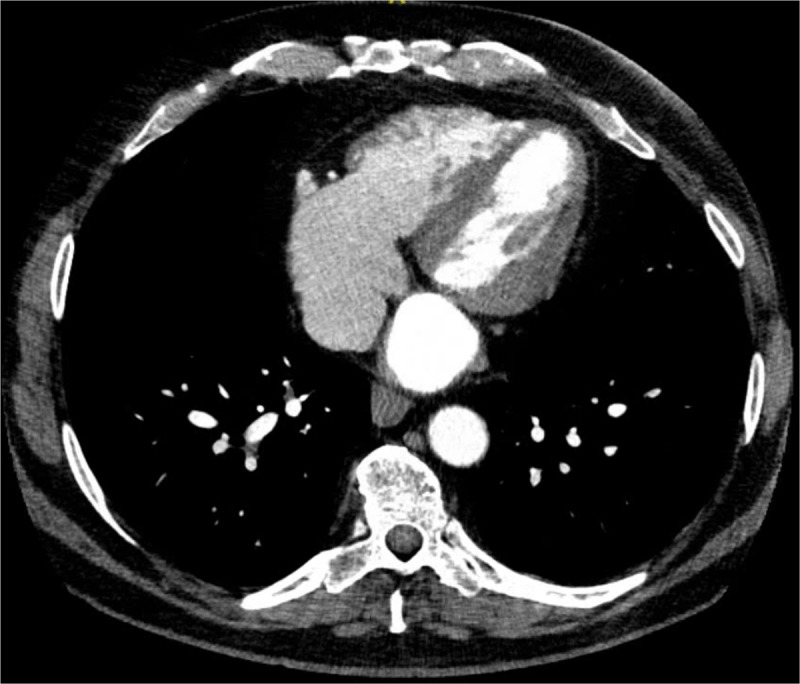
Computed tomography done prior to the atrial fibrillation ablation did not show a pseudoaneurysm.

The patient had mitral isthmus ablation 1 year prior to this presentation. In preparation for the ablation, the patient had a cardiac CT that did not show a pseudoaneurysm (***Figure 4***) or obstructive coronary artery disease.

Mitral isthmus ablation has been associated with spasm in the left circumflex artery.^1^ Therefore, it is likely that at the time of the ablation, the patient had prolonged spasm leading to a silent myocardial infarction with subsequent development of a pseudoaneurysm.

The patient underwent resection of a bilobed pseudoaneurysm and repair with placement of a Hemashield dacron patch (MAQUET Cardiovascular LLC). There was extensive expansion of the pseudoaneurysm beyond the initial point of rupture. The total diameter of the aneurysm was 7.2 cm and the orifice measured approximately 4 × 3 cm. CMR 1 week post repair showed a thin myocardial repair graft at the mid-inferolateral segment of the LV with mild biventricular dysfunction (***Video 7***).

**Video 7:** Cardiac magnetic resonance 1 week post repair showed a thin myocardial repair graft at the mid-inferolateral segment of the left ventricle with mild biventricular dysfunction, also at *https://youtu.be/AxCbNGwEZ6U*.

## Discussion

Ventricular pseudoaneurysm results from rupture of the myocardium that is walled off by pericardium, fibrous tissue, or hematoma. It lacks true myocardium or endocardium in its wall and typically has a narrow neck.^2^ Myocardial infarction, cardiac surgery, trauma, and infections are well-recognized causes of ventricular pseudoaneurysms. Of all cardiac surgeries, mitral valve replacement and aneurysmectomy carry the highest risk.^2,3^ The site of the pseudoaneurysm is primarily related to the etiology. Post-myocardial infarction pseudoaneurysms often occur in the inferior or posterolateral wall and less commonly at the apex. Postoperative pseudoaneurysms are usually in the posterior subannular region after mitral surgery, right ventricular outflow tract after congenital heart surgery, and in the subaortic region and/or aortomitral intervalvular fibrosa after aortic valve replacement.^3^

Meanwhile, endocarditis can be complicated by pseudoaneurysm at the aortomitral intervalvular fibrosa.^3^ On the contrary, “true aneurysms” have all layers of the myocardium but they are thinned out, scarred, or fibrotic. Additionally, the neck is wide and most commonly occurs at the ventricular apex or anterior wall.^4^ Our patient had a pseudoaneurysm related to a posterolateral infarction that likely resulted from spasm in the left circumflex artery during mitral isthmus ablation.^1^ To our knowledge, this is the first reported LV pseudoaneurysm resulting from mitral isthmus ablation.

Patients with LV pseudoaneurysms present with a wide spectrum of symptoms ranging from being completely asymptomatic to sudden cardiac death.^3^ On exam, 70% of patients will have a murmur, most likely to-and-fro in character.^2^ Almost all patients with pseudoaneurysm will have an abnormal ECG, most commonly in the form of nonspecific changes or an old infarct. However, 20% of patients will have ST-segment elevation.^2^ TTE is a reasonable first step for diagnosing pseudoaneurysms. Nonetheless, CT or CMR better define the extent of the aneurysm and can assist in surgical planning. Surgery is the preferred approach because medical management carries a 50% mortality risk, while surgical perioperative mortality is less than 10%.^2,5,6^ More recently, percutaneous closure has shown promising results in high-risk surgical candidates.^7^

## Key points

- Mitral isthmus radiofrequency ablation can cause injury in the left circumflex artery territory.
- Multimodality imaging is essential to determine the etiology and anatomy of pseudoaneurysms.
- Surgery is the treatment of choice in patients with ventricular pseudoaneurysms.

## CME credit opportunity

Houston Methodist is accredited by the Accreditation Council for Continuing Medical Education (ACCME) to provide continuing medical education for physicians.

Houston Methodist designates this enduring material for a maximum of .25 AMA PRA Category 1 Credit™. Physicians should claim only the credit commensurate with the extent of their participation in the activity.

Click to earn CME credit: *https://cvent.me/Qqoy2n*.
